# Gut microbiota-associated N-Acetyl-L-leucine mediates fructose-induced hyperuricemia *via* the P300-URAT1 pathway

**DOI:** 10.1038/s41538-026-00917-1

**Published:** 2026-06-04

**Authors:** Yingsheng Hu, Mengyu Zhou, Tianbo Xie, Min Wei, Kaiming Wang, Hua Wei, Fen Zhang, Zhihong Zhang

**Affiliations:** 1 State Key Laboratory of Food Science and Resources, Nanchang University, Nanchang, China; 2 Sino-German Joint Research Institute, Nanchang University, Nanchang, China; 3 Agricultural, Food and Nutritional Science, University of Alberta, Edmonton, AB Canada; 4 Department of Physiology, CEGIIR, University of Alberta, Edmonton, AB Canada; 5 Department of Food Science and Engineering, College of Life Science and Technology, Jinan University, Guangzhou, China; 6 Qian Xuesen Collaborative Research Center of Astrochemistry and Space Life Science, Institute of Drug Discovery Technology, Ningbo, China

**Keywords:** Diseases, Microbiology

## Abstract

Hyperuricemia (HUA), a metabolic disorder characterized by elevated uric acid (UA), is marked by a rising incidence and trend toward earlier onset, both strongly associated with fructose intake. However, the roles of gut microbiota and their metabolites remain unclear. This study employed a fructose-induced HUA mouse model and fecal microbiota transplantation (FMT) to assess the impact of fructose-altered gut microbiota on the microbial community structure and UA degradation capacity in recipient mice. Non-targeted metabolomics was then utilized to identify key metabolites, and the HUA-inducing effects and underlying mechanisms of the metabolite were further validated. The results confirmed that microbiota from fructose-induced HUA mice promoted HUA development in recipients, significantly increasing the abundance of *Bacteroidota* and reducing microbial UA degradation ability. Specifically, recipient mice exhibited disordered amino acid metabolism, primarily characterized by elevated N-Acetyl-L-leucine. This metabolite was subsequently shown to induce HUA by promoting UA synthesis through enhanced adenosine deaminase and xanthine oxidase activity, as well as inhibiting UA excretion *via* upregulation of the reabsorption transporter urate transporter 1 (URAT1) through E1A binding protein p300 (P300) regulation. These results highlight N-Acetyl-L-leucine as a pivotal gut microbiota-associated metabolite in fructose-induced HUA and offer new insights and potential targets for future interventions.

**Figure:**
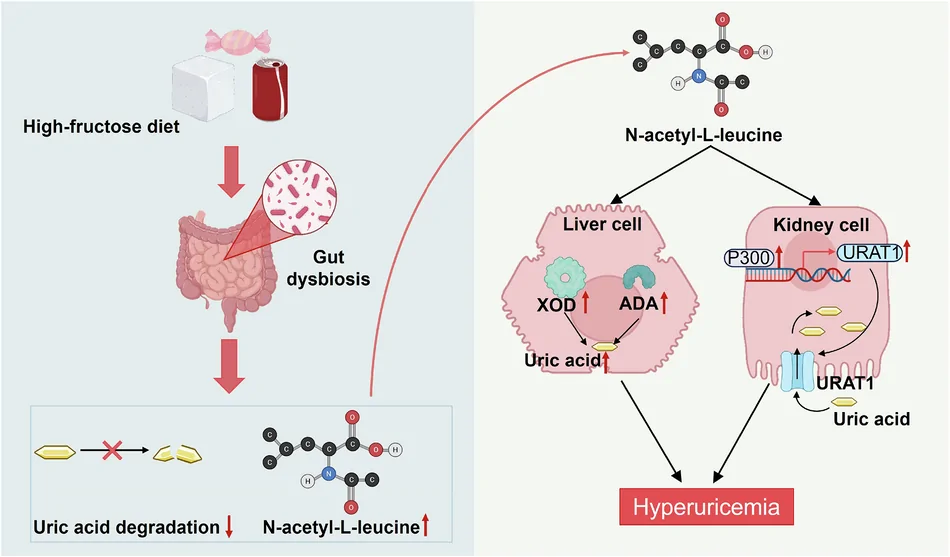

## Introduction

Hyperuricemia (HUA) is a metabolic disorder characterized by disrupted uric acid (UA) homeostasis, leading to elevated serum UA levels that surpass the normal physiological threshold^1^. Its rising incidence and trend toward younger population are closely linked to the dramatic dietary shifts over recent decades^2,3^. Modern diets, high in red meat, processed meats, and sugar-sweetened beverages^4^, directly introduce excessive exogenous purines and stimulate endogenous UA synthesis. Concurrently, the associated high intake of saturated fatty acids and critically low dietary fiber impairs renal UA excretion and exacerbates systemic inflammation^4^. These factors collectively drive the increased incidence of HUA, while the growing consumption of ultra-processed foods^5^ and sugar-sweetened beverages^6^ among younger individuals contributes to the earlier disease onset.

The pathogenesis of HUA primarily stems from the disorder of purine metabolism, manifesting as an imbalance between hepatic UA synthesis and its renal/intestinal excretion^7^. Unhealthy dietary patterns increase exogenous purine intake, elevate the activity of adenosine deaminase (ADA) and xanthine oxidase (XOD), and lead to increased UA production. These patterns also activate the inflammatory response, damaging renal^8^ and intestinal function and reducing UA excretion^9^. Notably, the increased consumption of free sugars, a hallmark of modern diets, has been closely associated with elevated UA levels. While fructose, the sweetest free sugar, is widely used in the food industry and commonly appears in the form of sucrose and high-fructose corn syrup (HFCS)^10^. Previous studies have shown that fructose induced HUA due to the absence of negative feedback regulation during its phosphorylation by ketohexokinase in the liver. This results in excessive ATP consumption, leading to the overproduction of AMP. AMP is then degraded into hypoxanthine, which ultimately breaks down into UA. This process results in abnormally elevated serum UA levels^11,12^. More recent evidence further indicates that fructose intake critically activates the pentose phosphate pathway, enhances de novo purine biosynthesis, and stimulates the XOD-mediated endogenous purine biosynthetic and metabolic pathways, collectively contributing to pathological UA accumulation^13^.

Gut microbiota dysbiosis also significantly contributes to the onset of HUA^14^, typically characterized by reduced microbial richness and diversity, along with an increased abundance of opportunistic pathogens^15^. Such dysbiosis disrupts the microbiota’s role in purine and UA metabolism, ultimately elevating UA levels. This is mechanistically evidenced by the significant enrichment of gut bacteria expressing xanthine dehydrogenase (which degrades purine into UA) and depletion of gut bacteria expressing uricase (which further degrades UA into urea)^16^. Simultaneously, elevated UA levels can induce chronic low-grade inflammation both systemically and within the gut^17^, thereby altering the intestinal microenvironment. These changes further affect the composition of the gut microbiota and the profiles of associated metabolites, creating a cycle that exacerbates HUA progression, which highlights the bidirectional and mutual relationship between gut microbiota and HUA^18^. Furthermore, studies have demonstrated that gut microbiota can ameliorate HUA through multiple mechanisms, including the degradation of UA^19^ and purines^20^ *via* specific gene clusters, activation of the UA transporter ATP Binding Cassette Subfamily G Member 2 (ABCG2) to promote intestinal UA excretion^21^, and the provision of energy to intestinal epithelial cells by specific metabolites (such as short-chain fatty acids) that help restore intestinal barrier function and modulate host immune responses^22,23^.

Although current research on fructose-induced HUA remains predominantly focused on hepatic purine metabolism pathways, the significant role of gut microbiota in HUA pathogenesis leads us to hypothesize that fructose also induces HUA by modulating gut microbiota composition and metabolite profiles. To test this, we first established a mouse model of HUA using a 10% fructose solution. We then employed fecal microbiota transplantation to confirm the essential role of the gut microbiota in this process. By integrating gut microbiome analysis with LC-MS-based non-targeted metabolomics, we further identified specific alterations in microbiota and metabolite compositions. The hyperuricemic effect of a key identified metabolite was subsequently validated both in vivo and in vitro. Moving beyond the conventional focus on hepatic purine metabolism, this study provides pivotal evidences that the gut microbiota and its metabolites are crucial mediators of fructose-induced HUA. These findings refine the mechanistic understanding of fructose-induced HUA and offer novel perspectives and potential targets for its future prevention and treatment.

## Results

### A high-fructose diet induces the onset of HUA within one week

**Fig. 1:**
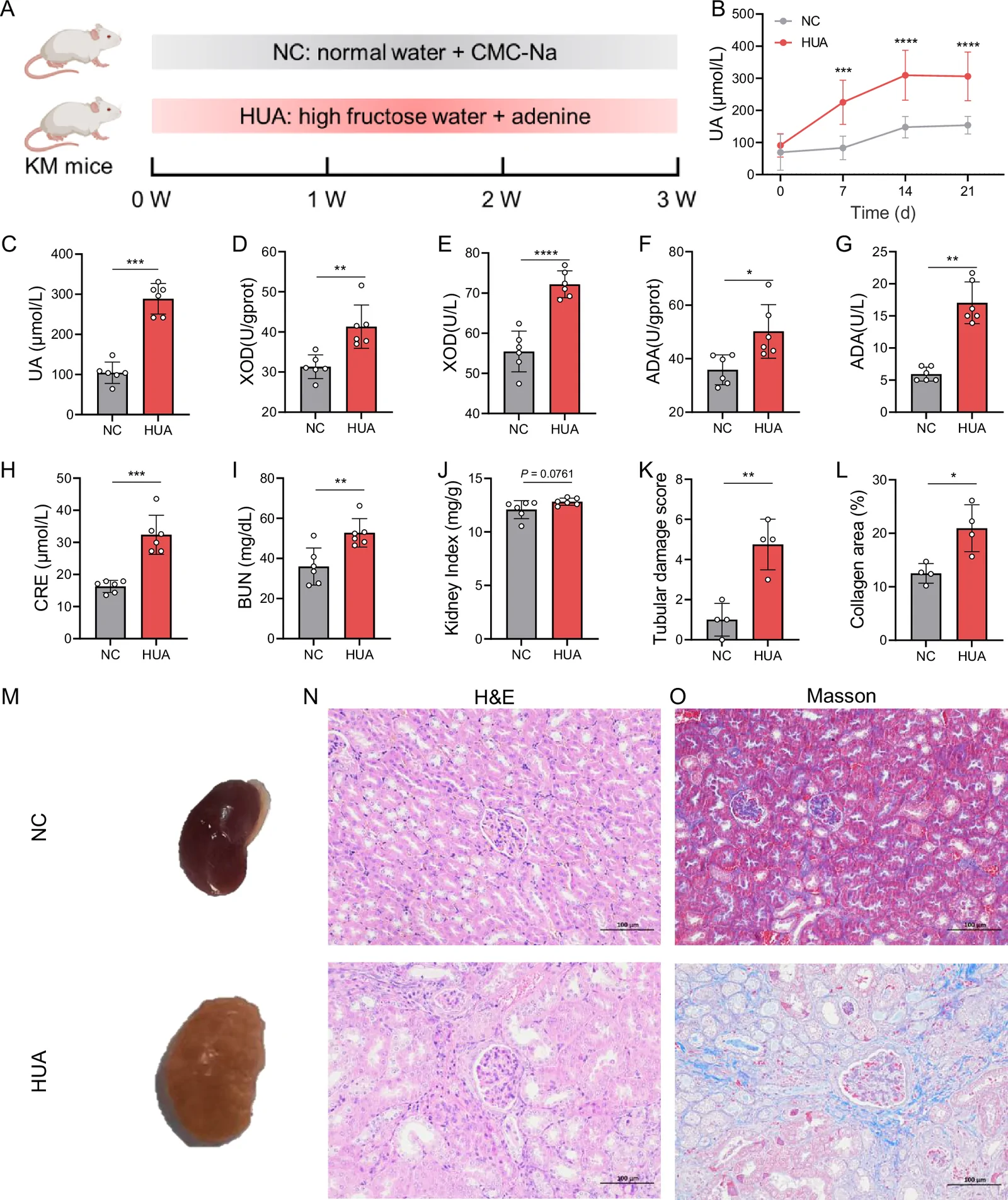
High fructose diet induces HUA in mice. **A** The animal experimental scheme; **B** Changes of serum UA in mice during the experimental period (*n* = 6); **C** Serum UA in mice at the end of the experiment (*n* = 6); Measurement of UA-producing enzyme activity (*n* = 6), including XOD in the liver (**D**) or serum (**E**) and ADA in the liver (**F**) or serum (**G**); Measurement of renal function indicators (*n* = 6), including CRE (**H**) and BUN (**I**); **J** Kidney index (*n* = 6); **K** Renal tubular damage score derived from H&E staining; **L** Renal fibrosis area based on Masson staining (*n* = 4); Representative images of mice kidney (**M**), kidney H&E staining (Black arrow: tubular dilatation; Blue arrow: luminal epithelial cell necrosis) (**N**) and kidney Masson staining (Red arrow: collagen fiber deposition) (**O**). Data are presented as mean ± SD. *p < 0.05; **p < 0.01; ***p < 0.001; ****p < 0.0001 by two-way ANOVA (**B**), unpaired *t* test (**C**–**F** and **I**–**L**), Mann–Whitney *U* tests (**G**) or Welch’s tests (**H**), vs NC group.

To validate the short-term effects of a high-fructose diet on HUA development, mice were administered a 10% fructose (w/v) solution ad libitum for three weeks to simulate human high-fructose consumption (Fig. 1A). Serum UA levels were significantly elevated after just one week of high-fructose intake and remained persistently higher than the control group throughout the experimental period (Fig. 1B, C, Fig. S1). Since UA is the end product of purine catabolism, we assessed the activity of key regulatory enzymes (XOD and ADA) in both liver and serum. The high-fructose diet significantly increased XOD and ADA activities in these samples (*p* < 0.05, Fig. 1D–G), indicating that high-fructose diet promotes UA production through accelerated purine metabolism.

Furthermore, persistent UA elevation resulted in abnormal changes in renal function, as reflected by significant increases in serum creatinine and blood urea nitrogen levels compared to CON group (Fig. 1H–I). Further histopathological examinations of kidney tissues revealed several structural abnormalities. Macroscopically, kidneys from HUA mice exhibited enlarged volume and a rough, irregular capsular surface (Fig. 1M). Histologically, H&E staining identified marked tubular dilatation and luminal epithelial cell necrosis (Fig. 1N), which was corroborated by significantly higher tubular damage scores (*p* < 0.01, Fig. 1K). Masson’s trichrome staining clearly demonstrated abnormal collagen fiber deposition in the renal tissue, indicating the development of fructose-induced renal fibrosis (Fig. 1L, O). Collectively, these results demonstrated that short-term high-fructose intake can rapidly induce HUA, likely through enhanced purine degradation and UA overproduction, which was associated with significant kidney damage and early fibrotic remodeling.

### FMT confirms that the altered gut microbiota from high-fructose diet induces HUA

**Fig. 2:**
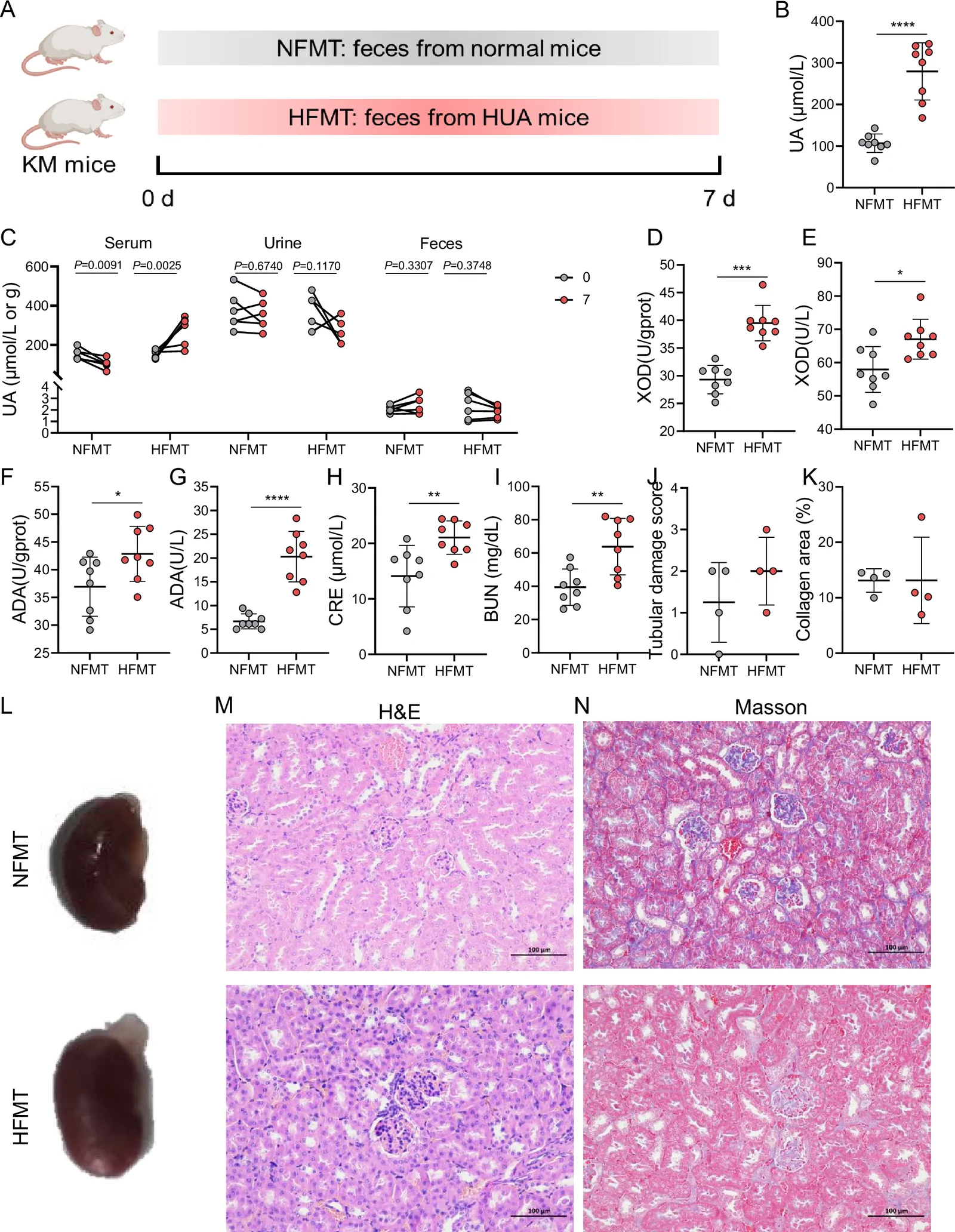
FMT from high-fructose diet-exposed mice induced HUA in normal mice. **A** The animal experimental scheme; **B** Serum UA in mice at the end of the experiment (*n* = 8); **C** Changes of serum, urine and feces UA in mice (*n* = 6); Measurement of UA-producing enzyme activity (*n* = 8), including XOD in the liver (**D**) or serum (**E**) and ADA in the liver (**F**) or serum (**G**); Measurement of renal function indicators (*n* = 8), including CRE (**H**) and BUN (**I**); **J** Renal tubular damage score derived from H&E staining (*n* = 4); **K** Renal fibrosis area based on Masson staining (*n* = 4); Representative images of mice kidney (**L**), kidney H&E staining (**M**) and kidney Masson staining (**N**). Data are presented as mean ± SD. *p < 0.05; **p < 0.01; ***p < 0.001; ****p < 0.0001 by unpaired *t* test (**B**–**C** and **F**–**K**) or Mann–Whitney *U* tests (**D**–**E**), vs NFMT group.

Human metagenomic studies have demonstrated that gut microbiota dysbiosis is a key feature in HUA development^24,25^. Given that dietary factors largely depend on the gut microbiome to modulate host physiology^26^, we hypothesized that high-fructose diets induce HUA primarily by reshaping gut microbiota composition. To test this, we transplanted fecal microbiota from donor mice fed a high-fructose diet (which developed HUA after three weeks) into antibiotic-treated recipient mice with depleted native microbiota (Fig. 2A). One week after FMT, recipient mice exhibited significantly elevated serum UA levels, recapitulating HUA phenotype observed in donors (Fig. 2B). In contrast, urinary and fecal UA excretion decreased in the recipient mice (Fig. 2C), suggesting that the fructose-altered gut microbiota may impair UA excretion. Moreover, both liver and serum analysis showed markedly increased activities of XOD and ADA in recipient mice (*p* < 0.05, Fig. 2D–G), indicating that the reshaped gut microbiota effectively enhanced the systemic UA biosynthesis capacity. Interestingly, these mice also displayed elevated serum creatinine and blood urea nitrogen levels (*p* < 0.01, Fig. 2H–I), suggesting functional renal dysfunction. However, systematic renal histopathological analyses (including H&E staining for tubular injury and Masson staining for fibrosis) revealed no evidence of typical renal injury or significant structural abnormalities (Fig. 2J–N). These results implied that fructose-remodeled gut microbiota contributed to HUA mainly by augmenting UA synthesis and limiting its excretion.

### Effects of high-fructose diets on gut microbiome structure and host metabolism

**Fig. 3:**
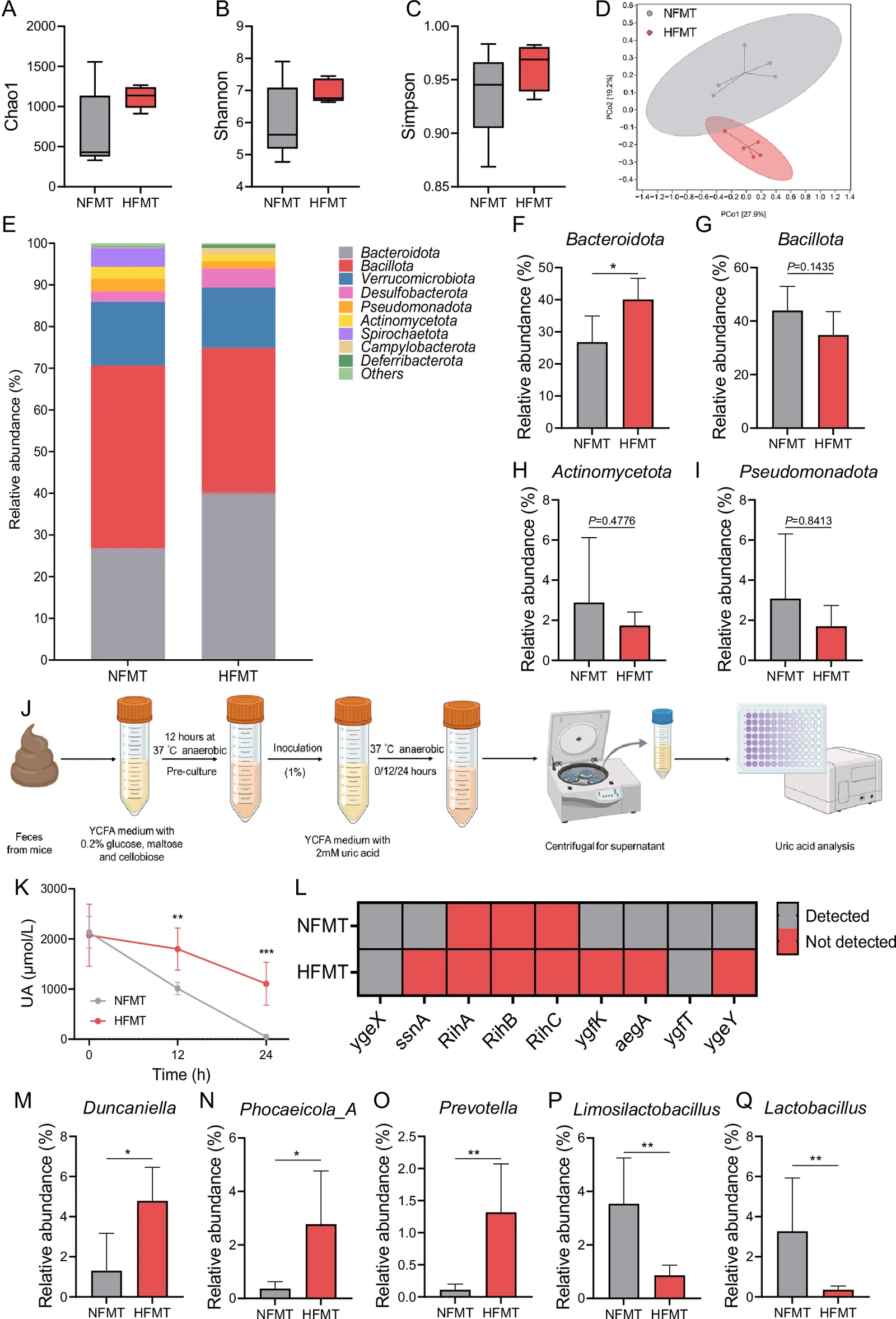
Alterations of gut microbiota in mice treated with FMT. α-diversity assessed through Chao1 (**A**), Shannon (**B**) and Simpson (**C**) index (*n* = 5); **D** β-diversity analysis by PCoA on Bray-Curtis distance; **E**–**I** The relative abundance of bacteria from cecum content at the phylum level (*n* = 5); **J** Schematic diagram of in vitro UA degradation experiment (*n* = 6); **K** UA detection in supernatant at different times; **L** Previously reported determination of UA and purine degradation gene clusters; **M**–**Q** The relative abundance of bacteria from cecum content at the genus level (*n* = 5). Data are presented as mean ± SD. *p < 0.05; **p < 0.01; ***p < 0.001 by Mann–Whitney *U* tests (**A**, **I** and **M**–**Q**), Welch’s tests (**B** and **H**), unpaired *t* test (**C**, **F**, and **G**) or two-way ANOVA (**K**), vs NFMT group.

Building on the FMT results confirming the gut microbiota’s pivotal role in high-fructose diet-induced HUA, we further characterized the microbial profiles of HFMT and NFMT mice using 16S rRNA gene sequencing and non-targeted LC-MS metabolomics. Although α diversity indices (Chao1, Shannon, and Simpson) showed no significant differences in species richness or evenness between the two groups (Fig. 3A–C). PCoA revealed a significant spatial separation of the gut microbiota (Fig. 3D), indicating that high-fructose induced gut microbiota dysbiosis is composition-driven rather than abundance-dependent.

At the phylum level, HFMT mice exhibited an increase abundance of *Bacteroidota*, which lack the capacity to consume UA^19^, alongside a reduction abundance of *Bacillota*, *Actinomycetota*, and *Pseudomonadota*, which are capable of consuming UA (Fig. 3E–I). This shift was confirmed by in vitro fecal UA degradation assays that gut microbiota from HFMT mice exhibited significantly reduced UA degradation capacity, along with the absence of UA-degrading gene clusters in gut bacteria (Fig. 3J–L). Genus-level analysis further identified enrichment of inflammation-associated genera (*Duncaniella* and *Phocaeicola_A*, and *Prevotella*) and depletion of the abundance of probiotic-associated genera (*Limosilactobacillus* and *Lactobacillus*) in the HFMT group (*p* < 0.05, Fig. 3M–Q). LEfSe analysis confirmed the expansion of potentially harmful bacteria such as *Duncaniella*, *QAMM01*, *Phocaeicola_A*, and *Prevotella* (Fig. S2). Collectively, these results suggest that high-fructose diets altered gut microbiota composition, thereby impairing UA degradation, and potentially exacerbating metabolic dysregulation through a disrupted intestinal microenvironment.

**Fig. 4:**
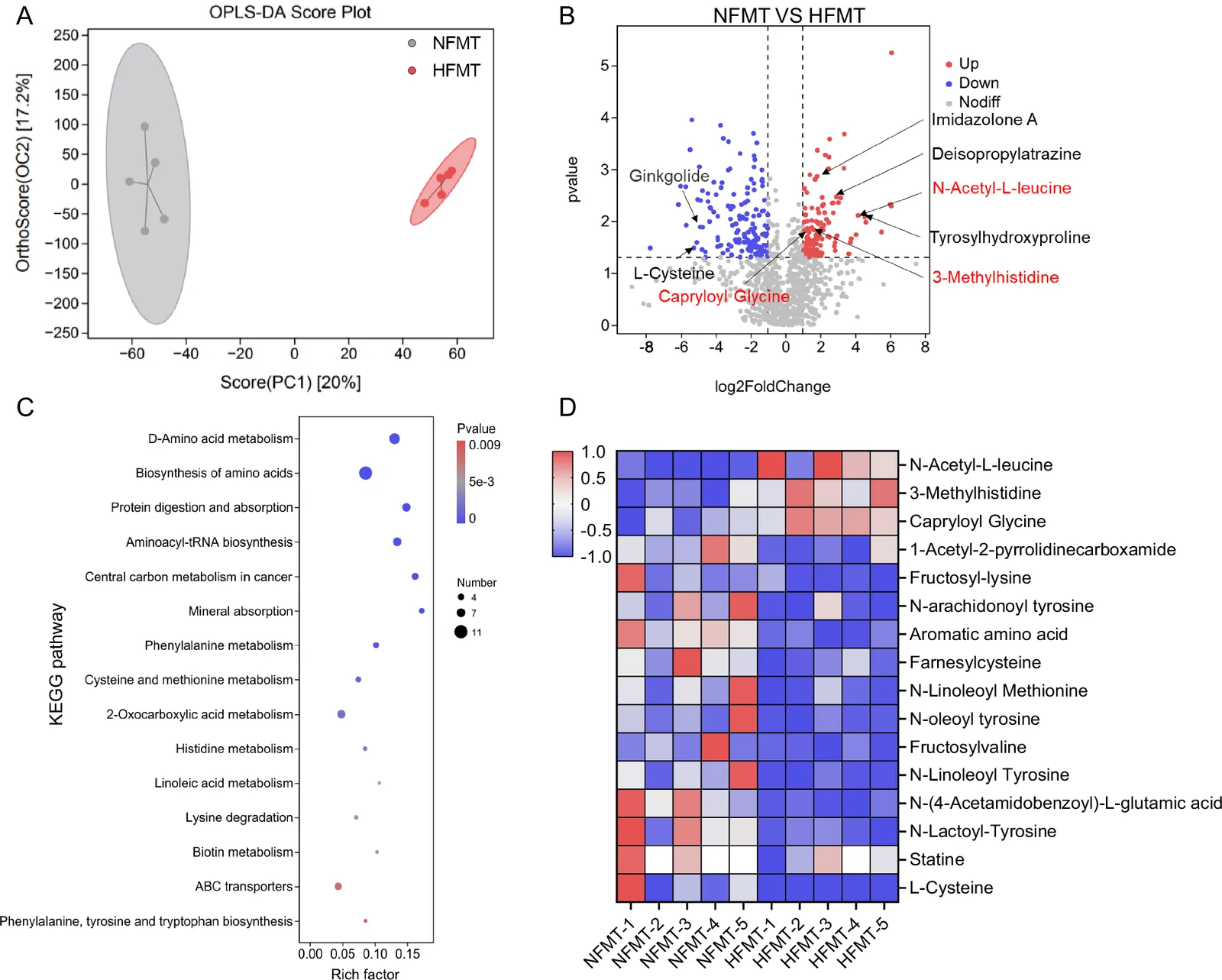
Alterations of gut metabolites in mice treated with FMT. **A** Score plot of orthogonal partial least squares discriminant analysis (OPLS-DA) between NFMT and HFMT groups; **B** Volcano plot of differential metabolites between NFMT and HFMT groups; **C** Metabolic pathway enrichment analysis of differential metabolites based on the Genomes (KEGG) database; **D** Heat map analysis of differential amino acids and their derivatives between NFMT and HFMT groups (*n* = 5).

As microbial metabolites are key mediators of host-microbiota crosstalk^27^ and SCFAs have been reported to alleviate HUA^28^, we then assessed the role of major SCFAs in HUA pathogenesis. However, no significant differences were observed between the two groups (Fig. S3A–D), suggesting high-fructose induced dysbiosis does not affect SCFAs production. In order to elucidate how high-fructose diets disrupt host metabolic networks by remodeling the gut microbiota, we employed non-targeted LC-MS metabolomics to systematically analyze changes in gut metabolites between HFMT and NFMT mice (Fig. S3F). Multivariate statistical analysis showed clear separation between the two groups (Fig. 4A; Fig. S3G–H). Differentially expressed metabolites showed that, compared to the NFMT group, the HFMT group exhibited significant accumulation of nephrotoxicity compounds (tyrosylhydroxyproline, desisopropylatrazine, and imidazolone A). Conversely, the levels of l-cysteine and ginkgolide, which possess potent antioxidant and anti-inflammatory activities, respectively, were significantly reduced (Fig. 4B). Furthermore, KEGG pathway enrichment analysis revealed that differentially expressed metabolites were significantly enriched in pathways associated with protein and amino acid metabolism and synthesis (Fig. 4C), consistent with prior studies implicating intestinal amino acid dysregulation in HUA progression^29^. Amongst the altered amino acids and derivatives (N-Acetyl-L-leucine, 3-methylhistidine, and capryloyl glycine), N-Acetyl-L-leucine showed the greatest upregulation in the HFMT group (Fig. 4D; Fig. S3E). Therefore, we propose that N-Acetyl-L-leucine is a key microbial metabolite mediating fructose-induced HUA.

### N-Acetyl-L-leucine induced HUA in mice through dual regulation of production and excretion

**Fig. 5:**
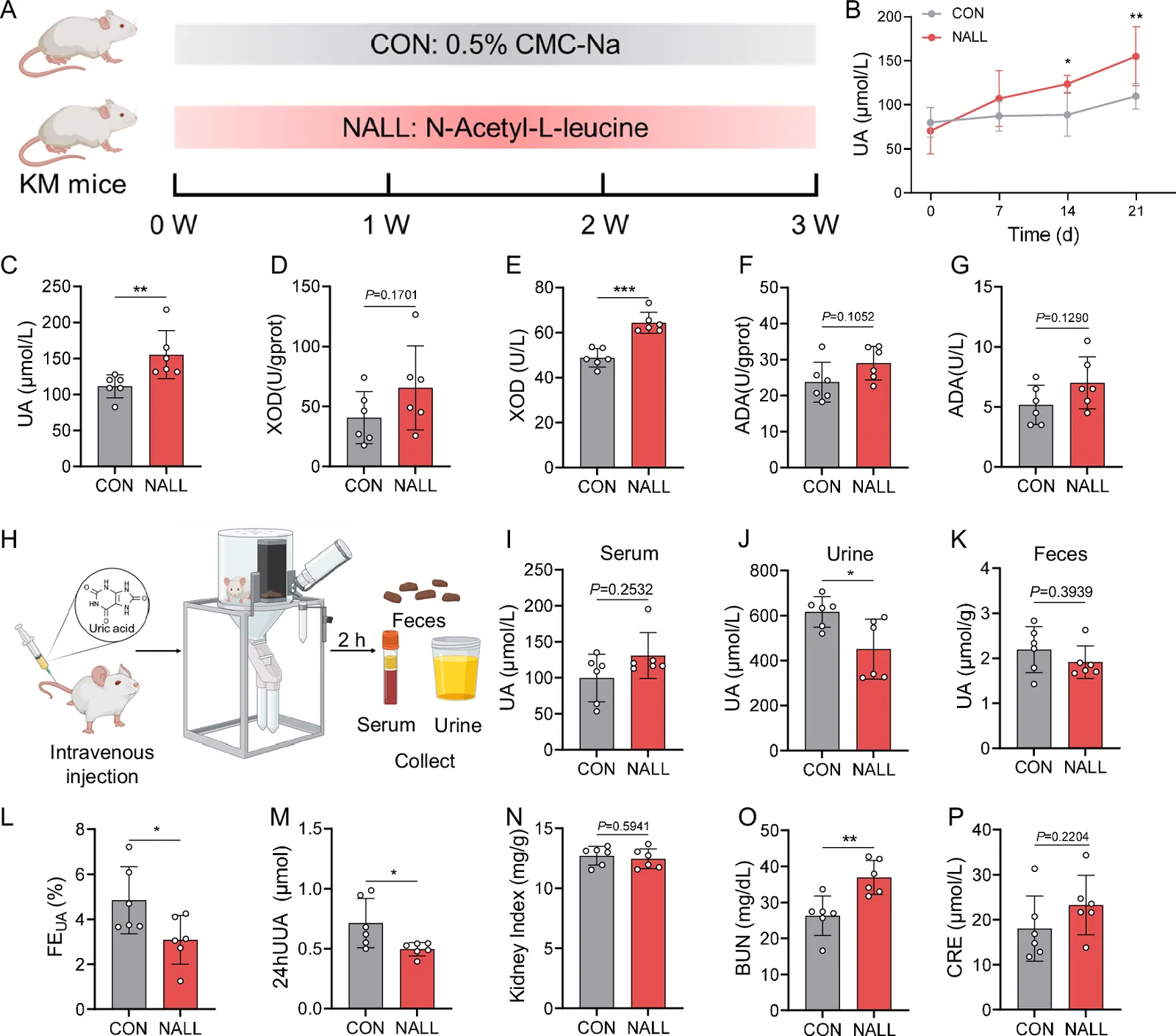
N-Acetyl-L-leucine induces hyperuricemia in mice. **A** The animal experimental scheme; **B** Changes of serum UA in mice during the experimental period (*n* = 6); **C** Serum UA in mice at the end of the experiment (*n* = 6); Measurement of UA-producing enzyme activity (*n* = 6), including XOD in the liver (**D**) or serum (**E**) and ADA in the liver (**F**) or serum (**G**); **H** Schematic diagram of intravenous injection experiment in mice; Determination of UA in serum (**I**), urine (**J**) and feces (**K**) of mice after intravenous injection of 100 μg of UA (*n* = 6); **L** Fractional urinary excretion of UA (n = 6); **M** Urinary excretion of urate during 24 h (*n* = 6); **N** Kindey index (*n* = 6); Measurement of renal function indicators (*n* = 6), including CRE (**O**) and BUN (**P**). Data are presented as mean ± SD. *p < 0.05; **p < 0.01; ***p < 0.001 by two-way ANOVA (**B**), Mann–Whitney *U* tests (C and **I**–**K**), Welch’s tests (**B** and **H**) or unpaired **t** test (**D**–**G** and **L**–**P**), vs CON group.

Based on metabolomic findings that identified N-Acetyl-L-leucine as a key candidate, we next directly evaluated its HUA-inducing effect in mice (Fig. 5A). Administration of N-Acetyl-L-leucine significantly elevated serum UA levels (*p* < 0.05, Fig. 5B, C), accompanied by an enhancement of key purine-catabolizing enzymes XOD and ADA activities (Fig. 5D–G). In particular, serum XOD activity exhibited a significant increase (*p* < 0.001, Fig. 5E), suggesting that N-Acetyl-L-leucine promotes UA production in vivo.

To determine the effect of N-Acetyl-L-leucine on UA excretion, we measured renal and intestinal excretion capacities in mice. The results demonstrated that N-Acetyl-L-leucine significantly inhibited renal UA excretion without affecting intestinal excretion (Fig. 5H–K). The urinary excretion fraction and 24 h urinary UA excretion results provided additional evidence for the inhibitory effect of N-Acetyl-L-leucine on renal UA excretion (*p* < 0.05, Fig. 5L–M). Interestingly, despite the diminished renal excretory capacity, no pathological renal injury was detected (Fig. 5N, Fig. S4). Furthermore, there were no substantial alterations observed in creatinine levels, which are indicative of renal function (Fig. 5P). Regarding the increase in blood urea nitrogen, N-Acetyl-L-leucine should be considered, as it is an acetylated amino acid derivative that may elevate the exogenous nitrogen load and potentially obscure true changes in renal function (Fig. 5O). Collectively, the results demonstrated that N-Acetyl-L-leucine induced HUA through dual regulation of UA production and excretion without causing direct kidney damage.

### N-Acetyl-L-leucine accelerates the production of UA *via* enhanced purine catabolism

**Fig. 6:**
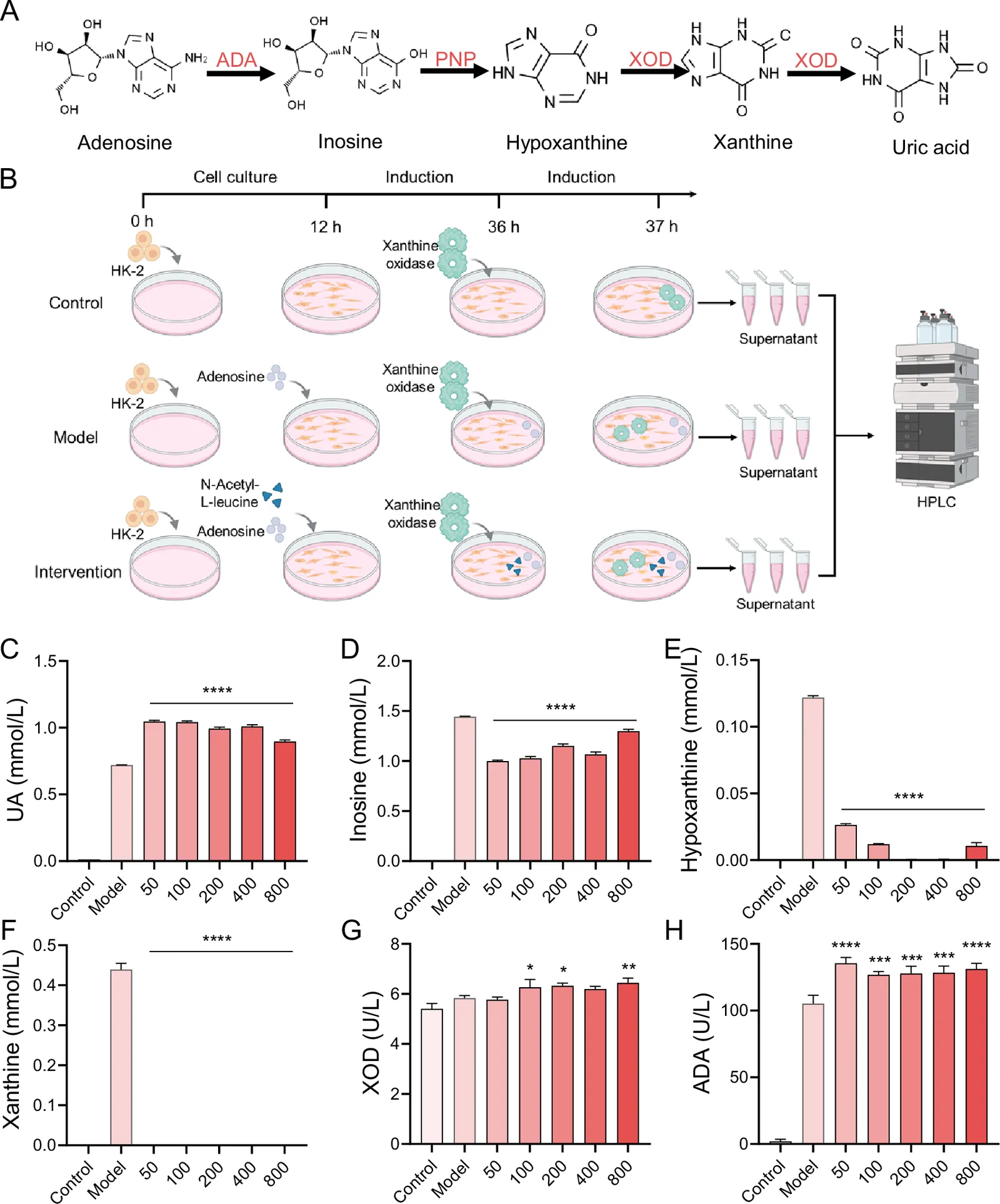
N-Acetyl-L-leucine accelerates UA production in human renal tubular epithelial cells. **A** Metabolic pathway of UA formation in HK-2 cells of this experiment; **B** Schematic representation of the experimental protocol; **C**–**F** Determination of UA and purine metabolism intermediate product concentrations (*n* = 3), including UA (**C**), Inosine (**D**), Hypoxanthine (**E**) and Xanthine (**F**); Determination of key enzyme activities in purine metabolism (*n* = 3), including XOD (**G**) and ADA (**H**). Data are presented as mean ± SD. *p < 0.05; **p < 0.01; ***p < 0.001; ****p < 0.0001 by one-way ANOVA, vs Model group.

We next sought to elucidate the underlying mechanism of N-Acetyl-L-leucine using an HK-2 cell model (Fig. 6A, B). The results showed that the levels of UA in the cell culture supernatant increased significantly (*p* < 0.0001, Fig. 6C) with treatment of N-Acetyl-L-leucine, confirming its direct role in UA synthesis. Then, we systematically measured the activity of key intermediates (inosine, hypoxanthine, and xanthine) and key rate-limiting enzymes (ADA and XOD) in purine metabolism pathway. The results demonstrated that N-Acetyl-L-leucine treatment significantly enhanced the activities of two key enzymes in the purine catabolism. (*p* < 0.05, Fig. 6G, H). Specifically, N-Acetyl-L-leucine markedly increased ADA activity, thereby accelerating the irreversible deamination of adenosine to inosine. Concurrently, it also potently activated XOD activity, which catalyzes the sequential oxidation of hypoxanthine to xanthine and finally to UA (*p* < 0.0001, Fig. 6E, F). This coordinated upregulation of both ADA and XOD effectively propels the substrate flux through the terminal steps of purine degradation, creating a pronounced drive for enhanced de novo UA synthesis. These data provided direct biochemical evidence that N-Acetyl-L-leucine serves as a metabolic regulator, amplifying the host’s intrinsic ability to produce UA. The results indicated that N-Acetyl-L-leucine significantly enhances the activities of ADA and XOD, thereby accelerating the conversion of adenosine to inosine and the transformation of hypoxanthine and xanthine into UA (Fig. 6C–H).

### N-Acetyl-L-leucine promotes UA reabsorption in HK-2 cells

**Fig. 7:**
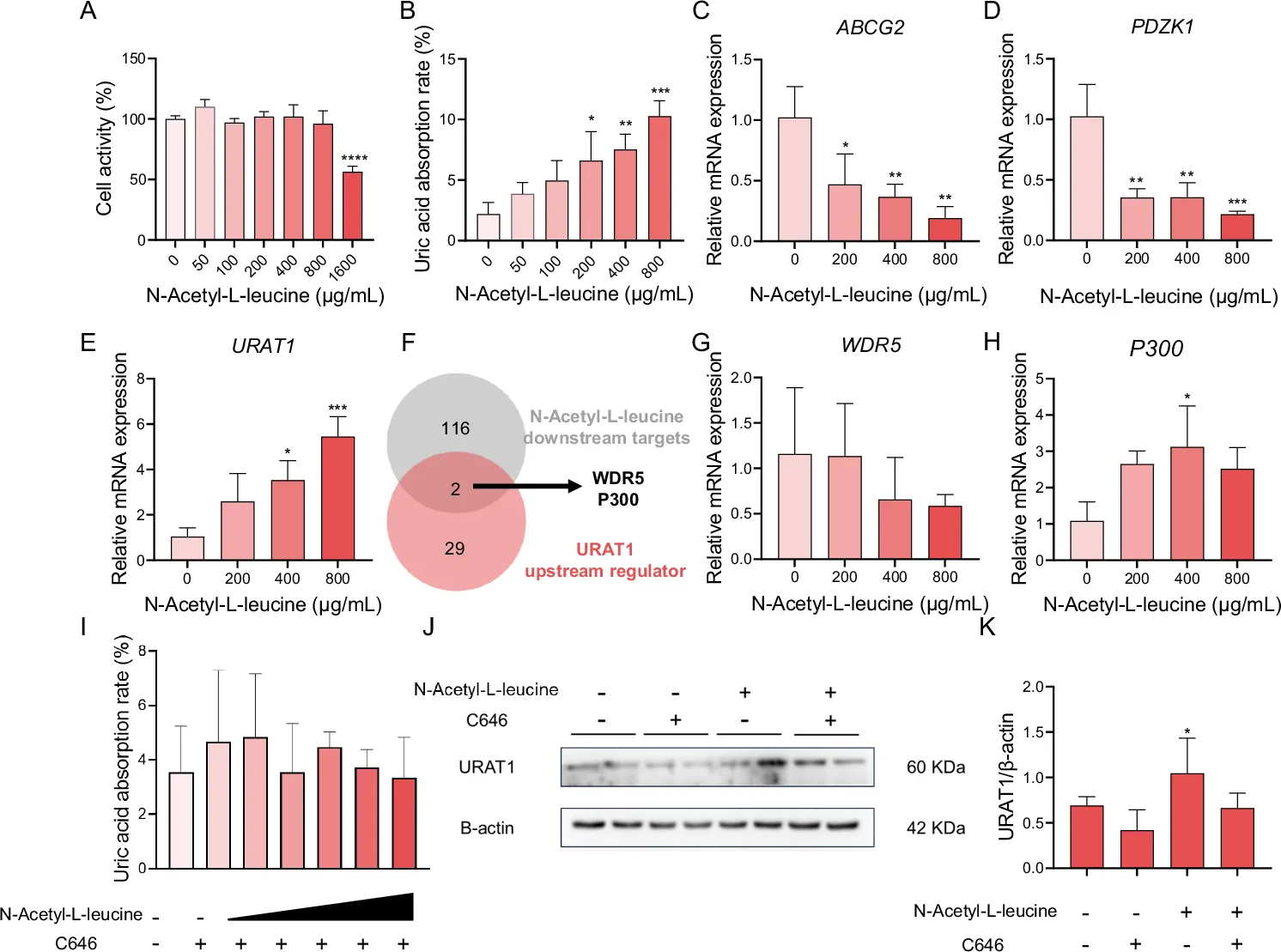
N-Acetyl-L-leucine promotes UA reabsorption in human renal tubular epithelial cells *via* the P300-URAT1 pathway. **A** Cell viability analysis in HK-2 cells treated with N-Acetyl-L-leucine (*n* = 3); **B** Measurement of UA absorption rate in HK-2 cells treated with N-Acetyl-L-leucine (*n* = 3); Relative expression of UA transport-associated protein (*n* = 3), including ABCG2 (**C**), PDZK1 (**D**) and URAT1 (**E**); **F** Venn diagram showing the screening strategy for the intersection of N-Acetyl-L-leucine downstream targets and URAT1 upstream regulator; **G**–**H** Relative expression of potential targets (*n* = 3); **I** Measurement of UA absorption rate in HK-2 cells treated with N-Acetyl-L-leucine and C646 (*n* = 3); **J**, **K** Western blot analysis of the expression of URAT1 (*n* = 3). Data are presented as mean ± SD. *p < 0.05; **p < 0.01; ***p < 0.001; ****p < 0.0001 by one-way ANOVA.

Additionally, we found that N-Acetyl-L-leucine treatment resulted in a dose-dependent increase in UA uptake (Fig. 7A, B), indicating a direct role in promoting UA reabsorption. Since renal tubular UA absorption is precisely regulated by the dynamic equilibrium between reabsorption and transport, we examined the expression of key UA transporters. N-Acetyl-L-leucine significantly downregulated the secretory transporters ABCG2 and PDZK1 while simultaneously upregulating the reabsorptive transporter URAT1 (Fig. 7C–E). This partially explains how N-Acetyl-L-leucine promotes UA absorption.

URAT1, a key protein in the UA reabsorption, which can mediate the counter-gradient uptake of UA from the lumen into renal tubular cells^30^, has been an important target in clinical application and research^31^. To elucidate the mechanism that how N-Acetyl-L-leucine upregulates URAT1, we predicted the downstream targets and upstream transcription factors of N-Acetyl-L-leucine and URAT1 using the compound-target prediction platform Super-PRED and human transcription factor database hTFtarget, respectively. Following this approach, we identified the epigenetic regulator WDR5 and the histone acetyltransferase P300 as potential targets. Experimental validation showed that N-Acetyl-L-leucine significantly promoted P300 expression, with no significant effect on WDR5 (Fig. 7F-H). This suggests that P300 mediates the regulation of URAT1 by N-Acetyl-L-leucine. To confirm the functional role of P300, we inhibited its activity using the specific antagonist C646. P300 blockade abolished the ability of N-Acetyl-L-leucine to promote UA reabsorption (Fig. 7I) and suppressed URAT1 upregulation(Fig. 7J-K). These results confirmed that N-Acetyl-L-leucine promotes the development of HUA by specifically increasing URAT1-mediated UA reabsorption through a P300-dependent transcriptional mechanism.

### High-fructose diets induce HUA *via* the P300-URAT1 pathway

**Fig. 8:**
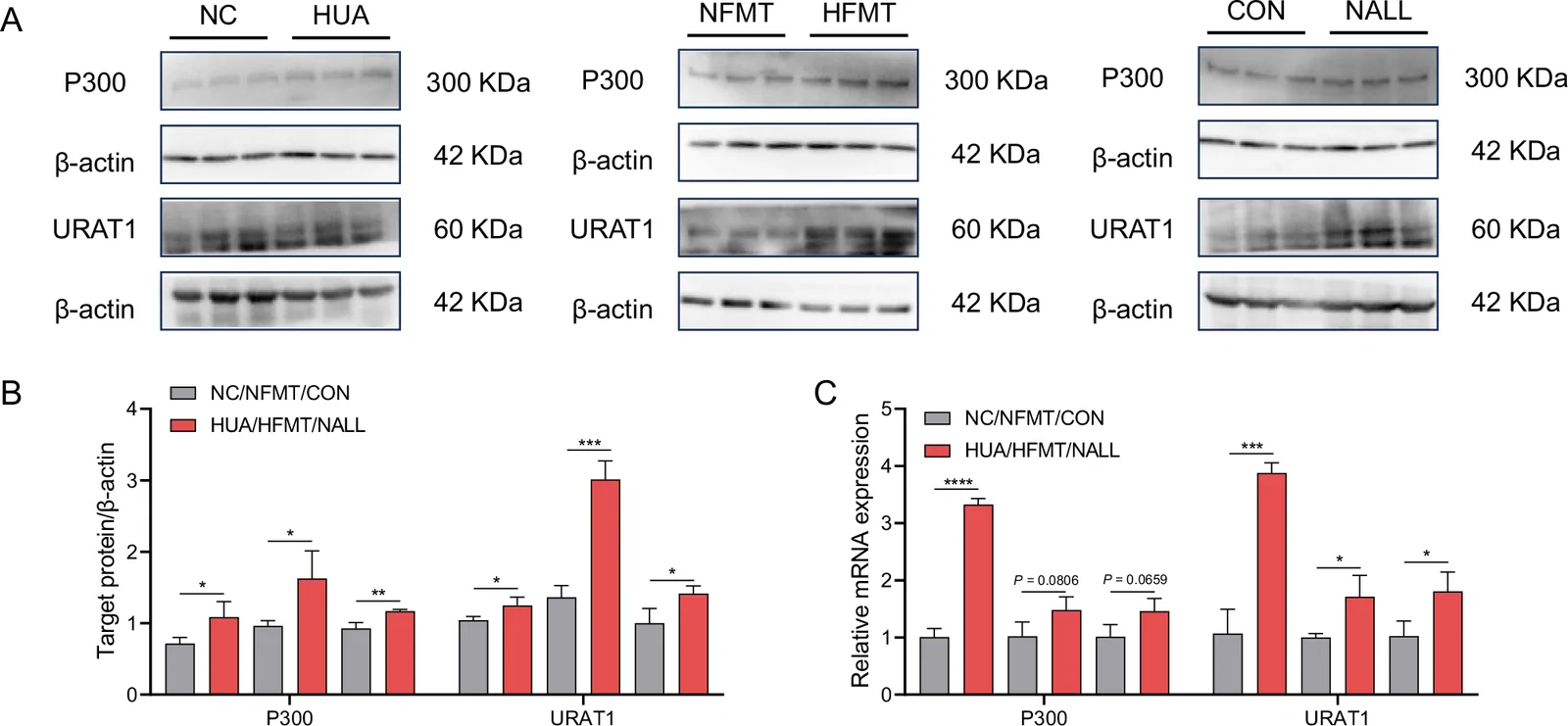
Activation of the P300-URAT1 Pathway. **A** Western blot of P300 and URAT1 in kidney; **B** Quantification of P300 and URAT1 in kidney (*n* = 3); **C** Relative expression of P300 and URAT1 in kidney (*n* = 3). Data are presented as mean ± SD. *p < 0.05; **p < 0.01; ***p < 0.001 by unpaired *t* test, vs NC/NFMT/CON group.

Building on our findings that N-Acetyl-L-leucine promotes UA reabsorption through P300-mediated URAT1 upregulation, we investigated whether this pathway is activated in vivo under high-fructose diet conditions. Protein and transcriptional levels analysis demonstrated consistent upregulation of P300 and URAT1 in mice fed a high-fructose diet, received fructose-remodeled gut microbiota, and treated with N-Acetyl-L-leucine (Fig. 8). This demonstrated that a high-fructose diet can induce HUA through the P300-URAT1 pathway.

## Dicussion

The rising consumption of fructose, especially through sugar-sweetened beverages, has been recognized as a significant risk factor for HUA^32^. This association stems from the unique metabolic pathway of fructose in mammalian systems, characterized by the absence of negative feedback regulation^33^. It is worthy noting that fructose metabolism is dependent on intestinal absorption and transportation through the portal venous system to reach the liver^11^, highlighting the crucial role of intestinal processes in fructose metabolism. Our study demonstrates that fructose-induced HUA can be transmitted through the gut microbiota *via* high-fructose diet and FMT. Through integrated microbiome sequencing, metabolomic profiling, and HK-2 cell models, we have identified N-Acetyl-L-leucine as a pivotal microbial metabolite mediating fructose-induced HUA. The present findings reveal a hitherto unrecognized mechanism and provide a novel perspective on the cognitive induction of HUA.

Using a mouse model with fructose-containing drinking water combined with a low dose of adenine, which can be endogenously induced by fructose, we successfully simulated human high-fructose consumption and ensuring a stable HUA phenotype. After just one week, the rapid elevation of uric acid levels, enhanced purine metabolism, and notable kidney damage observed underscore the pivotal role of fructose in triggering HUA^34,35^. Importantly, the finding that gut microbiota transplantation increased serum UA levels in healthy recipients aligns with prior work and indicates that the fructose-regulated gut microbiota can functionally transmit susceptibility across hosts^36^. Although our FMT experiment established the causal role of the gut microbiota, germ-free animal models would offer more definitive evidence and therefore warrant further investigation. The fructose diet also markedly increased the abundance of *Bacteroidota*. While an increased abundance of *Bacteroidota* is frequently associated with favorable metabolic outcomes in conditions such as obesity^37^, its correlation with HUA progression in our model appears contradictory. This observation can be understood from a functional perspective, as *Bacteroidota* are widely reported to lack the capability for UA degradation^19^. Therefore, their expansion might lead to a relative deficiency in intestinal UA catabolism under high-fructose conditions. a finding that aligns with prior cohort studies identifying elevated *Bacteroidota* as a characteristic gut microbiota feature in individuals with HUA and gout^38,39^.

It is well recognized that gut microbiota metabolites play key roles in host metabolism^27^. However, pinpointing their disease-specific functions remains challenging. Our findings help address this gap by further clarifying how fructose-induced dysbiosis contributes to HUA via these metabolites. Non-targeted metabolomics combined with KEGG pathway enrichment analysis revealed a severe disruption in the gut microbial metabolic profile under fructose intervention, with amino acid metabolism being the most significantly disturbed pathway. This finding aligns with prior HUA/gout metabolomic studies^40,41^, and specifically identified N-Acetyl-L-leucine as a key altered metabolite, underscoring the likely pivotal role of amino acid metabolic dysfunction in the pathogenesis of HUA.

The HUA-inducing effect of this key metabolite was subsequently validated in vivo. N-Acetyl-L-leucine significantly enhanced ADA and XOD activity, thereby accelerating UA production. Concurrently, renal excretion function was impaired, as reflected by reduced UA excretion fraction and diminished 24 h UA excretion, despite the absence of overt renal damage. In contrast to high-fructose feeding, N-Acetyl-L-leucine administration did not induce such structural kidney injury. This discrepancy may be explained by the fact that fructose itself, through its metabolism by ketohexokinase, generates oxidative stress and activates pro-inflammatory pathways, both of which can directly damage renal tissue^42^. This interpretation is further supported by our FMT results, in which recipient mice developed HUA but exhibited no significant kidney injury. These in vivo findings robustly confirm that N-Acetyl-L-leucine, a gut microbiota-associated metabolite induced by a fructose-rich diet, can simultaneously promote UA synthesis and inhibit its excretion.

Regarding the UA synthesis, N-Acetyl-L-leucine significantly elevated UA generation in the classical HK-2 cell HUA model (induced by adenosine and XOD)^43^. This effect stems from its enhancement of two key enzymes: ADA, which commits adenosine to purine degradation *via* deamination to inosine^44^, and XOD, a key rate-limiting enzyme that controls the sequential oxidation and hydroxylation to UA^45^. Collectively, this accelerates substrate flow through the purine catabolic pathway, thereby amplifying endogenous UA production. In addition, N-Acetyl-L-leucine exacerbates HUA by impairing renal UA excretion. This effect stems from a transcriptional reprogramming of key UA transporters: suppressing the efflux transporters ABCG2 and PDZK1 while potently upregulating URAT1. Since URAT1 is a major determinant of serum UA levels^46,47^, its induction provides a direct mechanism for reduced UA clearance. To elucidate how this metabolite regulates URAT1 transcription, we identified the transcriptional coactivator P300 and WDR5 as its critical upstream target^48^. Specifically, N-Acetyl-L-leucine selectively enhanced P300 expression. Subsequent functional studies using a specific P300 inhibitor (C646) confirmed its centrality, as inhibition completely reversed the metabolite’s effects on UA uptake and URAT1 expression. Therefore, our findings suggest that P300 is involved in the regulation of URAT1 expression, mediating the effect of N-Acetyl-L-leucine on renal gene expression. Future studies are warranted to determine whether this involves direct P300 recruitment to the URAT1 promoter or histone acetylation at this locus. Collectively, our findings establish N-Acetyl-L-leucine and its downstream P300-URAT1 axis as critical targets, offering a novel and precise approach for the dietary and therapeutic management of fructose-induced HUA.

**Fig. 9:**
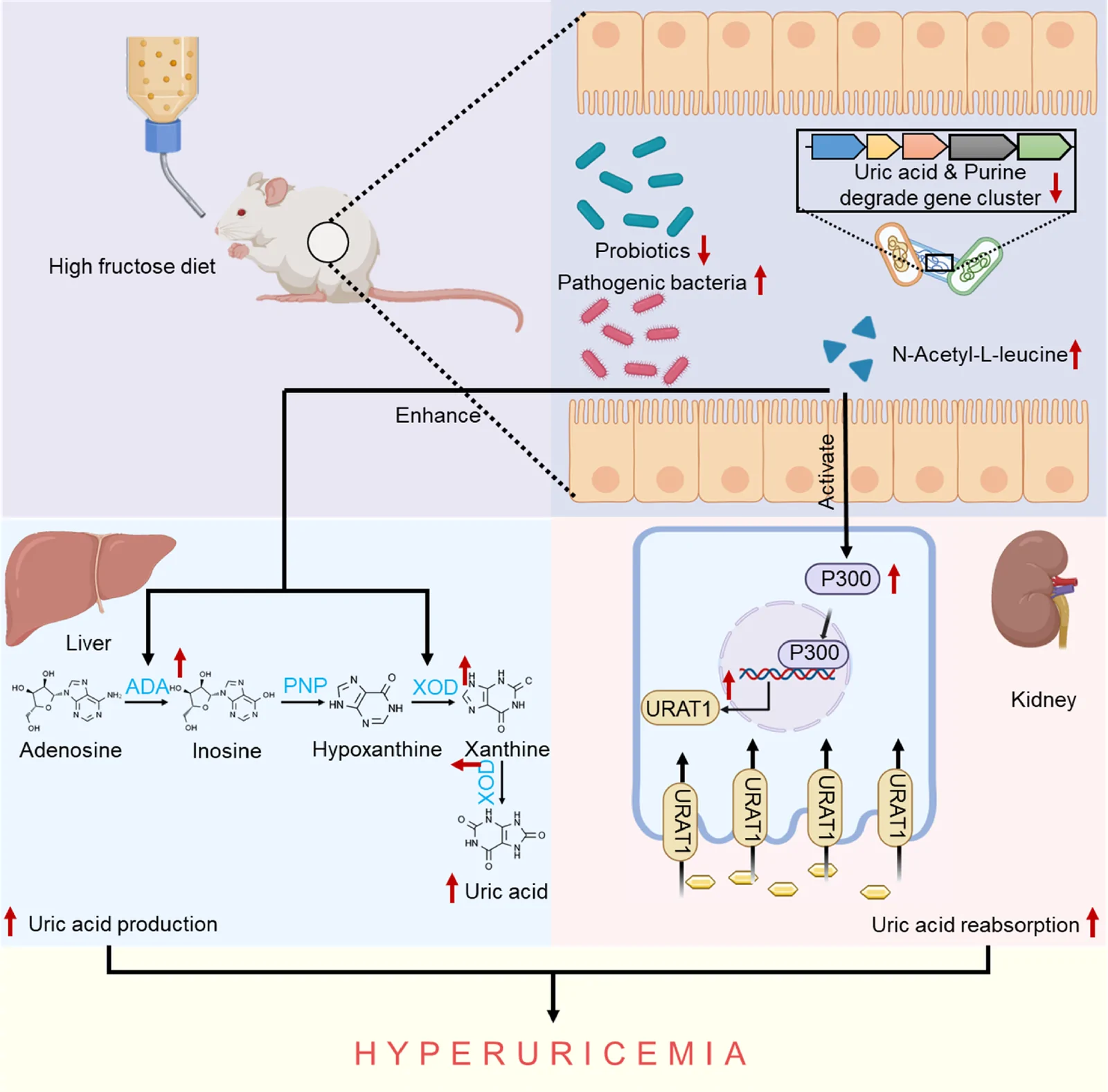
Mechanism of HUA induced by high-fructose diets *via* gut microbiota. The image was created in Biorender.com.

In conclusion, fructose-rich diet can induce HUA by disrupting the gut microbiota structure and metabolism. High fructose intake induces an imbalance in the ratio of commensal to potential pathogens, along with a significant deficiency in gene clusters previously implicated in UA and purine metabolism. Moreover, high-fructose diets perturb intestinal amino acid metabolism, as demonstrated by elevated N-Acetyl-L-leucine levels. This disruption accelerates endogenous UA production by enhancing ADA and XOD activity, thereby promoting the irreversible deamination of adenosine to inosine and the sequential oxidation and hydroxylation of hypoxanthine to xanthine and UA. Meanwhile, the activation of the transcription factor P300 upregulates the UA reabsorption protein URAT1, thereby promoting UA reabsorption and contributing to the development of HUA (Fig. 9). This study reveals a novel mechanism by which dietary fructose induces HUA through the gut microbiota and its metabolites, thereby establishing a theoretical foundation for precision intervention strategies based on dietary regulation and microbiota reprogramming.

## Methods

### Mice and diet

Male Kunming mice (6 weeks old) were purchased from SLAKE Jingda Laboratory Animal Co., Ltd. (Certificate No.: SCXK-2019-0004, Hunan, China). Experiments were conducted according to the protocol approved by Wuhan Myhalic Biotechnology Co., Ltd. Animal Experimentation Ethics Committee (HLK-20240617-002). All mice were held under standard conditions with an average temperature of 24°C and a 12 h light-dark cycle. Anesthesia was not required for gavage or tail vein injection procedures. For euthanasia, mice were exposed to gradual-fill CO₂ inhalation (30–70% cage volume/min from compressed gas), followed by cervical dislocation.

For the high-fructose-induced HUA mouse model, all mice were randomly assigned to either the high-fructose diet group (HUA, *n* = 6) or the control group (NC, *n* = 6) after a one week of adaptation period with free access to water. Following a previous method with modifications^49^, the HUA group consumed 10% (w/v) fructose solution and received daily gastric administration of 200 μL of 50 mg/kg adenine to accelerate and sustain UA elevation for three weeks, thereby establishing the HUA model. The NC group consumed normal water and received daily gastric administration of 200 μL of 0.5% CMC-Na.

For the FMT experiment, with reference to a previous method^50^, recipient mice were administered 200 μL of an antibiotic cocktail (vancomycin, 100 mg/kg; neomycin sulfate, 200 mg/kg; metronidazole, 200 mg/kg; and ampicillin, 200 mg/kg) *via* daily oral gavage for one week to remove their native gut microbiota. Subsequently, all recipient mice were randomly assigned to either the high-fructose diet recipient group (HFMT, *n* = 8) or the normal diet recipient group (NFMT, *n* = 8). The HFMT group received fecal supernatant from HUA group mice for one week, while the NFMT group received fecal supernatant from NC group mice over the same period. Throughout the one-week FMT period, all recipient mice were maintained on normal drinking water and standard chow, without any fructose or adenine supplementation, to ensure that any observed phenotypic changes could be attributed solely to the transplanted microbiota. Preparation of fecal supernatant is shown below: collect donor mouse feces daily, resuspend 100 mg in 1 mL of pre-chilled sterile PBS, vortex vigorously for 10 seconds. Then, centrifuge at 800 g for 3 min at 4 °C. Collect the supernatant for gastric lavage, and the whole process must be completed within 15 min.

In the N-Acetyl-L-leucine intervention mouse experiment, all mice were randomly assigned to a control group (CON, *n* = 6) and an N-Acetyl-L-leucine intervention group (NALL, *n* = 6) after one week of acclimation with free access to water. The CON group received daily oral administration of a 0.5% CMC-Na solution (200 μL), while the NALL group received a daily oral administration of a 0.5% CMC-Na solution containing 100 mg/kg N-Acetyl-L-leucine (200 μL) for three weeks.

### Mice renal function indicator assay

Before sacrificing the mice, blood samples were collected from their eyeballs. The blood was placed in a refrigerator at 4 °C to allow for natural sedimentation. Then, it was centrifuged at 1699 × g for 5 min to separate the serum. The supernatant was collected. Kits (Nos. C012-2-1, C011-2-1, C013-2-1) from Nanjing Jiancheng Bioengineering Institute were used to detect serum UA, creatinine, and blood urea nitrogen (BUN) levels to assess for changes in renal function.

### Assay of UA-related enzyme activity in mice

To assess changes in UA production capacity in mice, the activity of XOD and ADA in serum and liver tissue was measured according to the kit requirements provided by Nanjing Jiancheng Bioengineering Institute (A002-1-1, A048-2-1). Liver samples were processed into a 10% liver homogenate, and the supernatant obtained by centrifugation at 4 °C, 956 × *g* for 10 min was used for measurement. Protein content in the supernatant was determined using the BCA Protein Concentration Assay Kit (Biyun Tian, P0012S), and enzyme activity was analyzed per unit of protein.

### Analysis of kidney tissue injury and fibrosis

After sacrifice, both kidneys were collected, rinsed with pre-chilled PBS to remove residual blood, dried with filter paper, and precisely weighed to calculate the kidney-to-body-weight ratio (kidney weight in mg/body weight in g × 100%). The kidneys were fixed in 4% paraformaldehyde and embedded in paraffin. Sections of 5 mm thickness were prepared for hematoxylin and eosin (H&E) staining and Masson’s trichrome staining, with representative images captured at 200 × magnification. H&E staining images were evaluated by two independent reviewers. Tubular damage score was assigned according to published scoring systems^51^: obvious expansion of renal tubules with flattened cells (1 point), tubular type (2 points), exfoliated and necrotic cells in the lumen of renal tubules with no tubular type or cell debris (1 point), granular degeneration of epithelial cells (1 point), vacuolar degeneration (1 point), nuclear pyknosis (1 point). Masson-stained images were analyzed using Image-Pro Plus 6.0 software to quantify collagen fiber area.

### Gut microbiota analysis

The acquisition, processing, and sequencing of mouse cecal samples were performed as previously described^52^. Raw sequence data undergoes decoding using the demux plugin, followed by primer removal *via* the cutadapt plugin. Subsequently, the DADA2 plugin performs quality filtering, denoising, assembly, and chimera removal on the sequences. The obtained sequences were grouped based on 100% sequence similarity, yielding feature-based ASVs and abundance data. Subsequently, diversity indexes were calculated using QIIME2 software. Beta diversity analysis was performed using the UniFrac distance metric to investigate variations in the structure of microbial community among samples. These variations were visualized through principal coordinate analysis (PCoA).

### Metabolites analysis with LC-MS

During the process of mouse dissection, cecal contents were collected from at least five mice per group under low-temperature conditions. Samples were weighted accurately to 50 mg and added to 500 μL of pre-chilled methanol (containing 5 ppm 2-chlorophenylalanine). Subsequently, two steel balls were added to each sample for vortexing (30 s) and grinding for 120 s at 55 Hz, followed by ultrasonic disruption for 10 min at 500 W. Samples were then centrifuged at 4 °C and 15,294 × *g* for 10 min. After filtration through a 0.22 μm filter tip, the prepared samples underwent non-targeted metabolomics analysis using a high-resolution LC-MS/MS platform (Thermo Fisher Scientific) according to the previously described methodology^53^.

### Quantification of N-Acetyl-L-leucine in mice serum by HPLC

HPLC analysis was performed on an Agilent 1260 HPLC system equipped with an InfinityLab Poroshell 120 EC-C18 column (4.6 × 150 mm, 4 μm), using a diode array detector (DAD) set to monitor at 210 nm for the N-Acetyl-L-leucine in the mice serum. Mobile phase A consisted of 10 mM dibasic sodium phosphate, adjusted to a pH of 2.1 ~ 2.3 with phosphoric acid. Mobile phase B was acetonitrile. The chromatographic conditions were as follows: a constant flow rate of 0.6 mL/min; a column oven temperature of 30 °C; an injection volume of 10 μL; gradient elution; and a total run time of 20 min. Prior to gradient elution, the system was equilibrated for over 30 min at a flow rate of 0.6 mL/min with a composition of 95% mobile phase A and 5% mobile phase B. Mice serum samples were mixed with acetonitrile in a 1: 2 ratio to precipitate proteins. After thorough vortex mixing, the mixture was centrifuged at 15,000 × *g* for 10 min at 4 °C. The supernatant was collected, filtered through a 0.22 μm filter, and used for HPLC analysis.

### Determination of the effects of N-Acetyl-L-leucine on UA metabolism in mice

After fasting for 12 h, the mice received a 100 μg tail vein injection of UA and were transferred to metabolic cages for individual housing immediately. Urine and fecal samples were collected over the subsequent two hours, during which time food and water were withheld. After two hours, the mice were sacrificed, and blood was collected from the eyeball, and then the serum was separated from the blood (see Fig. 5H). The urine samples were placed in a 60 °C water bath to fully dissolve the UA. Fecal samples were weighed, mixed with 1 mL of PBS, and heated in a 60 °C water bath for 10 min. The samples were then vigorously shaken to break down the fecal matter and ensure complete dissolution. The mixture was then centrifuged at 106 × g for 5 min, and the supernatant was collected. UA levels in the serum, urine, and feces were measured according to the protocol for the UA assay kit (No. C012-2-1) from the Nanjing Jiancheng Bioengineering Institute.

### Assessment of UA excretion capacity in mice following N-Acetyl-L-leucine intervention

To quantify the effect of N-Acetyl-L-leucine on UA excretion capacity, mice were placed in metabolic cages after three weeks of N-Acetyl-L-leucine intervention and were fasted but allowed free access to water with 24 h urine collection. Blood samples were then obtained *via* eyeball puncture, and the serum was separated from the blood. Serum and urinary UA and creatinine levels were measured using the UA Assay Kit (No. C012-2-1) and the Creatinine Assay Kit (No. C011-2-1) from Nanjing Jiancheng Company, respectively. UA excretion capacity was assessed *via* the UA excretion fraction (FE_UA_) and 24 h UA excretion (24 h UUA). FEUA is calculated as follows: (urinary UA × serum creatinine) / (serum UA × urinary creatinine). 24 h UUA is calculated as follows: 24 h urine volume × urinary UA.

### HK-2 cell culture and activity assay

Human kidney tubular epithelial cells, Human Kidney-2 (HK-2), were cultured in RPMI 1640 medium supplemented with 10% fetal bovine serum, 100 KU/L penicillin G sodium salt, and 100 mg/L streptomycin sulfate at 37 °C in a humidified incubator with 5% CO₂. Cells were seeded at a density of 1 × 10⁴ cells /well in a 96-well plate and cultured overnight. After cells adhered, the spent medium was aspirated, and RPMI 1640 containing different concentrations of N-Acetyl-L-leucine (0, 50, 100, 200, 400, 800, 1600 μg/mL) was added. After 12 h incubation, remove the spent medium and wash twice with Hank’s Balanced Salt Solution (HBSS) buffer. Subsequently, 100 μL RPMI 1640 and 10 μL Cell Counting Kit-8 (CCK-8) reagent were added to each well. The mixture was incubated at 37 °C in the dark for 2 h. Cell viability was determined by measuring absorbance at 450 nm.

### Establishment of a HUA model of HK-2 cells

Based on the method of Hou et al. with modifications^43^, HK-2 cells were seeded at a density of 1 × 10⁵ cells per well in a 12-well plate. After 12 h of culture in RPMI 1640 medium, the old medium was discarded. Then, the control group received fresh RPMI 1640 medium, while the model group received RPMI 1640 medium supplemented with 2.5 mM adenosine, and the intervention group received the model medium supplemented with varying concentrations of N-Acetyl-L-leucine. After 24 h, 0.005 U/mL XOD was added to all groups. Following a further 1 h incubation period, the culture supernatants were collected for HPLC analysis.

### HPLC analysis of purine and UA in the HK-2 cell HUA model

HPLC analysis was performed on an Agilent 1260 HPLC system equipped with an InfinityLab Poroshell 120 EC-C18 column (4.6 × 150 mm, 4 μm), using a DAD set to monitor at 254 nm for the presence of purines and UA in the HK-2 cell supernatant. Mobile phase A consisted of 0.52 mM sodium 1-pentanesulfonate and 0.20 M potassium dihydrogen phosphate, adjusted to a pH of 4.0 with phosphoric acid. Mobile phase B was a 9:1 (v/v) mixture of mobile phase A and chromatographic-grade acetonitrile. The chromatographic conditions were as follows: a constant flow rate of 0.6 mL/min; a column oven temperature of 35 °C; an injection volume of 10 μL; gradient elution; and a total run time of 25 min. Prior to gradient elution, the system was equilibrated for over 30 min at a flow rate of 0.6 mL/min with a composition of 95% mobile phase A and 5% mobile phase B.

### Effects of N-Acetyl-L-leucine on UA reabsorption in HK-2 cells

HK-2 cells were resuspended in RPMI 1640 medium containing different concentrations of N-Acetyl-L-leucine (0, 50, 100, 200, 400, 800 μg/mL) and seeded at a density of 1 × 10⁵ cells/well in a 96-well plate. After 12 h of incubation, the medium was removed and 200 μL of RPMI 1640 medium containing 1.5 mmol/L UA stock solution was added to each well. After incubating in the incubator for 30 min, 5 μL of sample solution was aspirated from each well. The remaining UA concentration was determined using the UA kit (C012-2-1, Nanjing Jiancheng Biological Engineering Institute). The UA absorption rate (%) was calculated as follows: UA absorption rate (%) = [(UA concentration in stock solution - Remaining UA concentration) × 100%] / UA concentration in stock solution.

### In silico analysis for downstream targets of hippuric acid and the upstream transcriptional regulator of URAT1

N-Acetyl-L-leucine was used to predict its potential downstream targets by Super-PRED (https://prediction.charite.de, SMILES: CC(C)C[C@@H](C( = O)O)NC( = O)C, with probability > 50% and prediction accuracy > 70%). A total of 118 potential downstream targets of N-Acetyl-L-leucine were found. As for the transcriptional regulator of urate transporter 1 (URAT1), hTFtarget (https://guolab.wchscu.cn/hTFtarget, genome cordinate: chr11: 64590641-64602353) was employed, and a total of 31 potential upstream regulators of URAT1 were found.

### Relative expression of UA transporter in HK-2 cells

HK-2 cells were resuspended in RPMI 1640 medium containing various concentrations of N-Acetyl-L-leucine (0, 200, 400, and 800 μg/mL) and seeded at a density of 2 × 10⁵ cells per well in a 6-well plate for 12 h incubation. The cells were then treated with the Trizol kit to extract cellular RNA, which was subsequently reverse transcribed into cDNA using the Takara reverse transcription kit (Takara, Japan), following the instructions. The reaction systems were prepared with the Takara SYBR Green Fluorescent Quantitative PCR Kit. qPCR analysis was performed using reverse-transcribed cDNA as a template, with β-actin protein serving as the internal reference gene to determine the relative expression levels of the uric acid secretory transporters ABCG2 and PDZ domain containing 1 (PDZK1) and the reabsorptive transporter URAT1. The primers used are listed in Supplementary Table.

### Western blot analysis of UA transporter

Total proteins were extracted from HK-2 cells and kidney tissues using a radioimmunoprecipitation assay (RIPA; Servicebio, Wuhan, China) buffer containing a cocktail of protease inhibitors. The total protein concentration was quantified by the BCA method to adjust the loading amounts for subsequent analysis. The extracted proteins were then subjected to 10% SDS-PAGE electrophoresis and transferred to an ethanol-activated, 0.45 µm PVDF membrane. Membranes were blocked and incubated overnight at 4 °C with primary antibodies, including URAT1 (1:3000, Proteintech), E1A binding protein p300 (P300) (1:750, Proteintech), and β-actin (1:5000, Epizyme Biotech). Following primary antibody incubation, membranes were treated with species-specific HRP-conjugated secondary antibodies at room temperature for one hour. Finally, protein bands were visualized using ultra-sensitive ECL chemiluminescent reagents on a chemiluminescence imaging system and quantified using ImageJ software.

### Statistical analysis

All experiments were performed at least three times and analyzed statistically using GraphPad Prism software (version 9.5). The experimental data are expressed as the mean ± standard deviation. To compare means between two groups, *t*-tests, Mann–Whitney *U* tests, Welch’s tests, or Kolmogorov–Smirnov tests were selected based on the normality of the distribution and the equality of the variances. For comparisons involving three or more groups, one-way ANOVA followed by Dunnett’s tests was performed. Two-way ANOVA with Sidak’s test was used to determine the difference of UA at the different time points. Differential metabolites of metabolomics results were identified using a combination of Student’s *t*-test (*p* < 0.05) and fold change (FC) > 2. *p* < 0.05 indicates statistical significance.

## Supplementary information

Supplementary material

## Data Availability

The data that support the findings of this study are available in Zenodo at https://doi.org/10.5281/zenodo.18335189.
