# Supplementary material for: Gut microbiota-associated N-Acetyl-L-leucine mediates fructose-induced hyperuricemia via the P300-URAT1 pathway
Source: NPJ Sci Food. 2026 Jun 4;10:271. doi: 10.1038/s41538-026-00917-1 (PMC13550416; doi:10.1038/s41538-026-00917-1)
Supplement: Supplementary file 1 [file 41538_2026_917_MOESM1_ESM.pdf]

Supplementary material

Supplementary Table of primer

Gene name	Sequence (5'→3')
h-PDZK1-F	CAGCCTCACATTCTTCTT
h-PDZK1-R	GGTCACAACTCATTCCTT
h-ABCG2-F	AATACATCAGCGGATACTA
h-ABCG2-R	AATAAGCCACCATCATAAG
h-URAT1-F	ACACAGCCAGTCTCTTGATGGAGTG
h-URAT1-R	CCGTGATGAGCCAGCGTGCC
h-WDR5-F	AGTGCCTCAAGACTTTGCCAGC
h-WDR5-R	CGATGAGCGTCTTCAGGCACTG
h-P300-F	AGCCAAGCGGCCTAAACTC
h-P300-R	TCACCACCATTGGTTAGTCCC
h-βactin-F	ACCGGGCATAGTGGTTGGA
h-βactin-R	ATGGTACACGGTTCTCAACATC

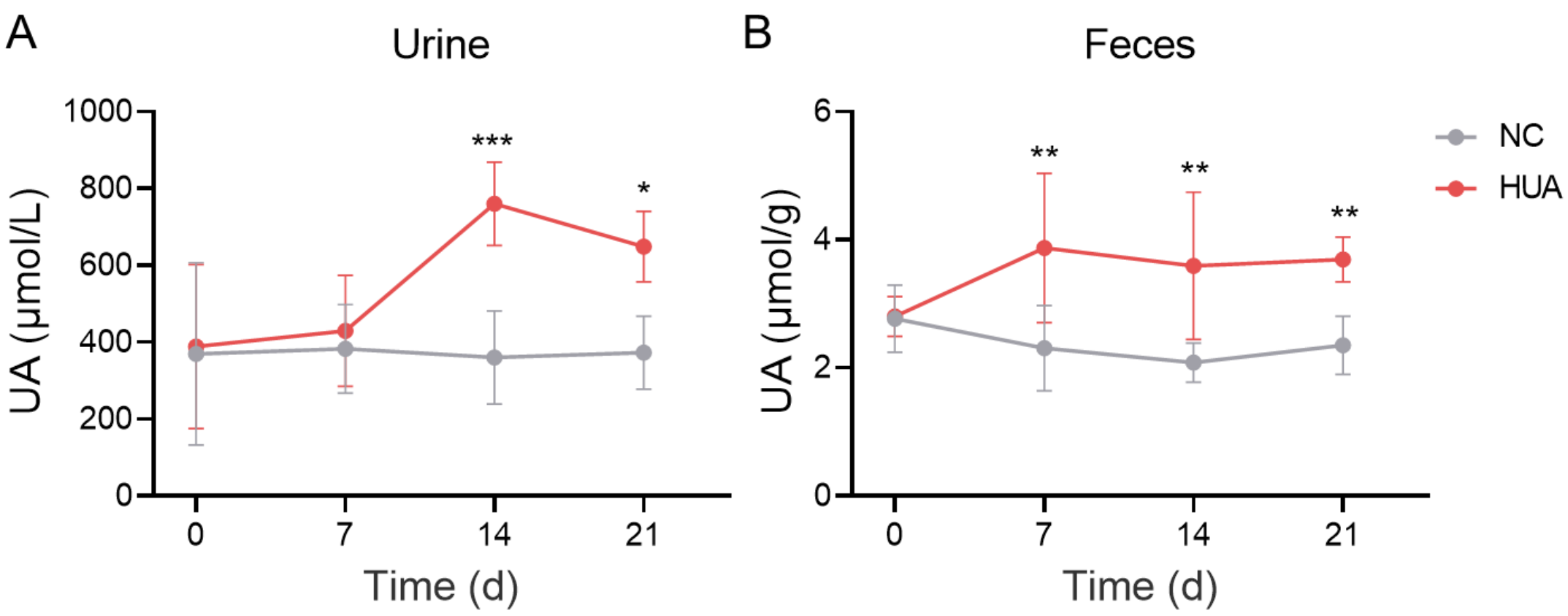

Fig. S1 Monitoring of UA in mice during the experiment. (A) Changes of urine UA in mice during the experimental period; (B) Changes of feces UA in mice during the experimental period. Data are presented as mean ± SD. *$p < 0.05$; **$p < 0.01$; ***$p < 0.001$ by two-way ANOVA, vs NC group.

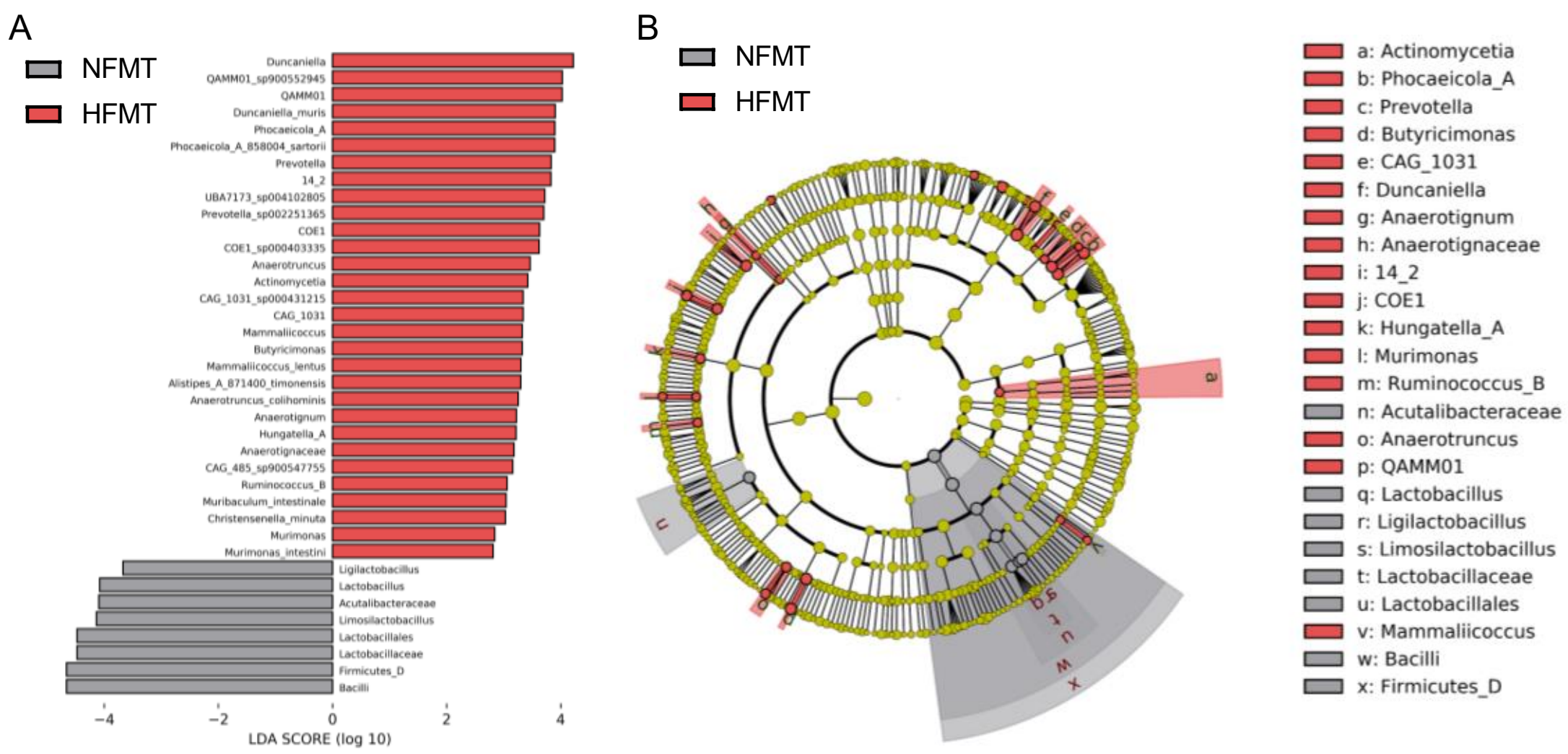

Fig. S2 LEfSe analysis of gut microbiota. (A) LDA of the most differential taxa between the NFMT and HFMT groups; (B) A cladogram of the differential taxa of cecum microbiota.

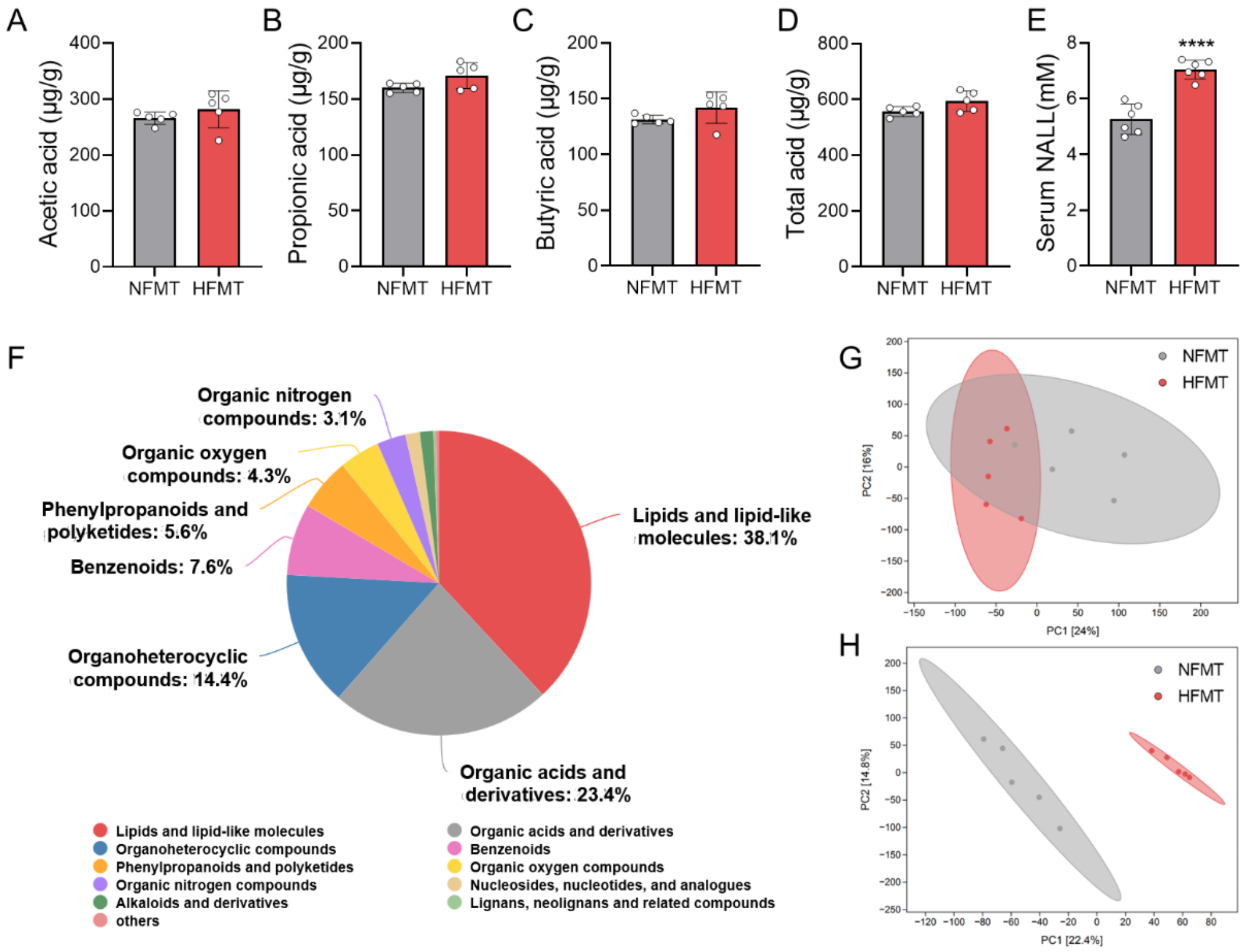

Fig. S3 Detection of metabolites in mouse cecum. (A-D) SCFAs in mouse cecum, including acetic acid (A), propionic acid (B), butyric acid (C) and total acid (D); (E) N-Acetyl-L-leucine in mice serum; (F) Classification of differential metabolites between NFMT and HFMT mice; (G) Score plot of principal component analysis (PCA) between NFMT and HFMT groups; (H) Score plot of partial least squares discrimination analysis (PLS-DA) between NFMT and HFMT groups. Data are presented as mean ± SD. ****$p < 0.0001$ by unpaired t test, vs NFMT group.

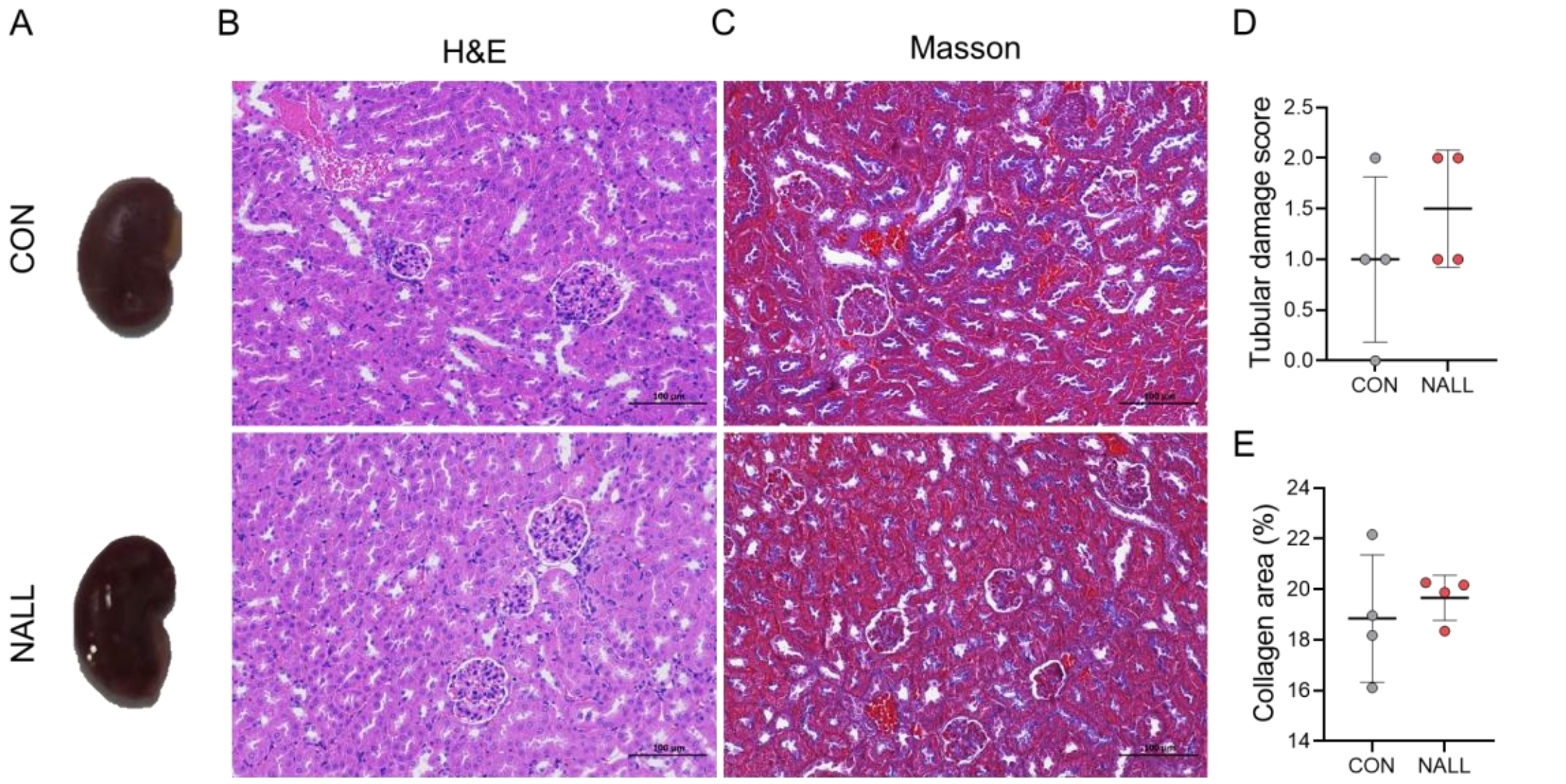

Fig. S4 Mice kidney pathology analysis. (A-C) Representative images of mice kidney (A), kidney H&E staining (B) and kidney Masson staining (C); (D) Renal tubular damage score derived from H&E staining; (E) Renal fibrosis area based on Masson staining. Data are presented as mean ± SD.

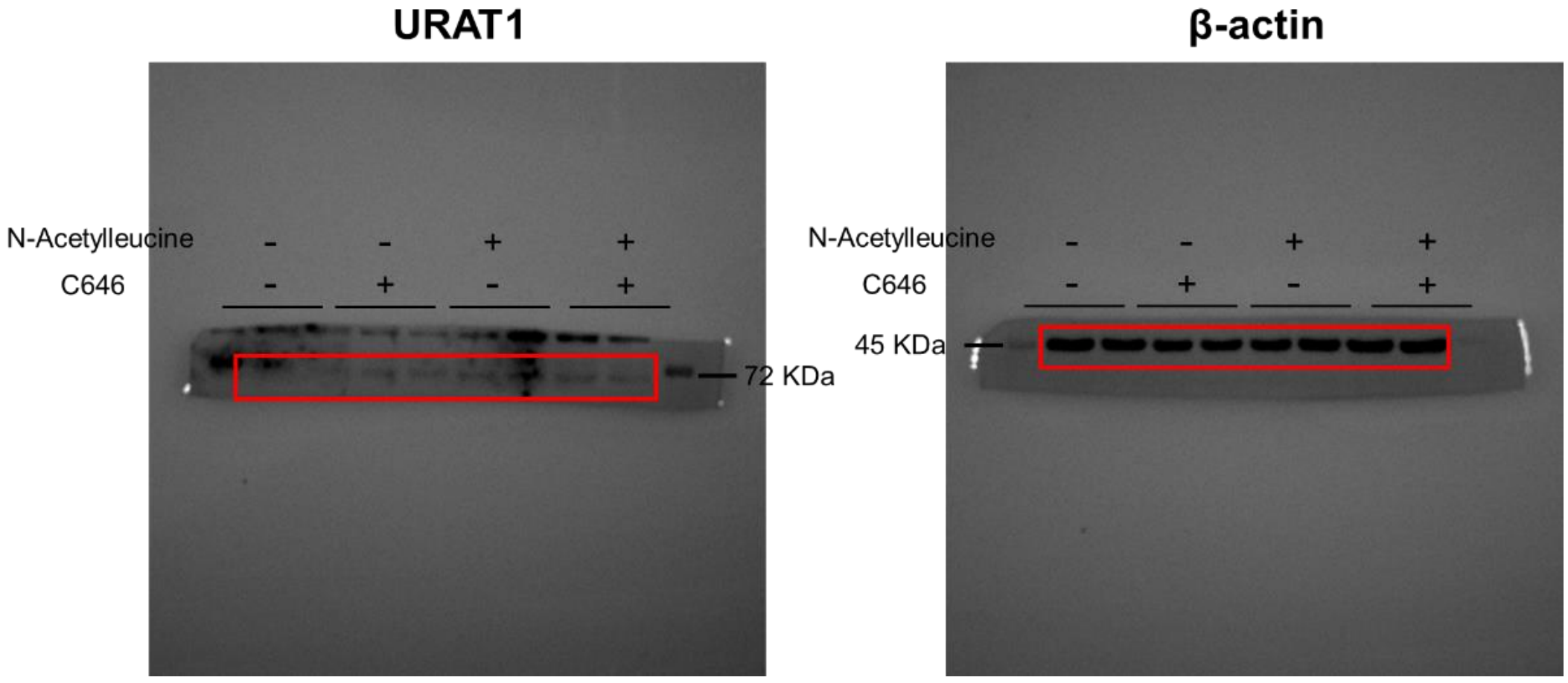

Fig. S5 Uncropped images of blots for Fig. 7J.

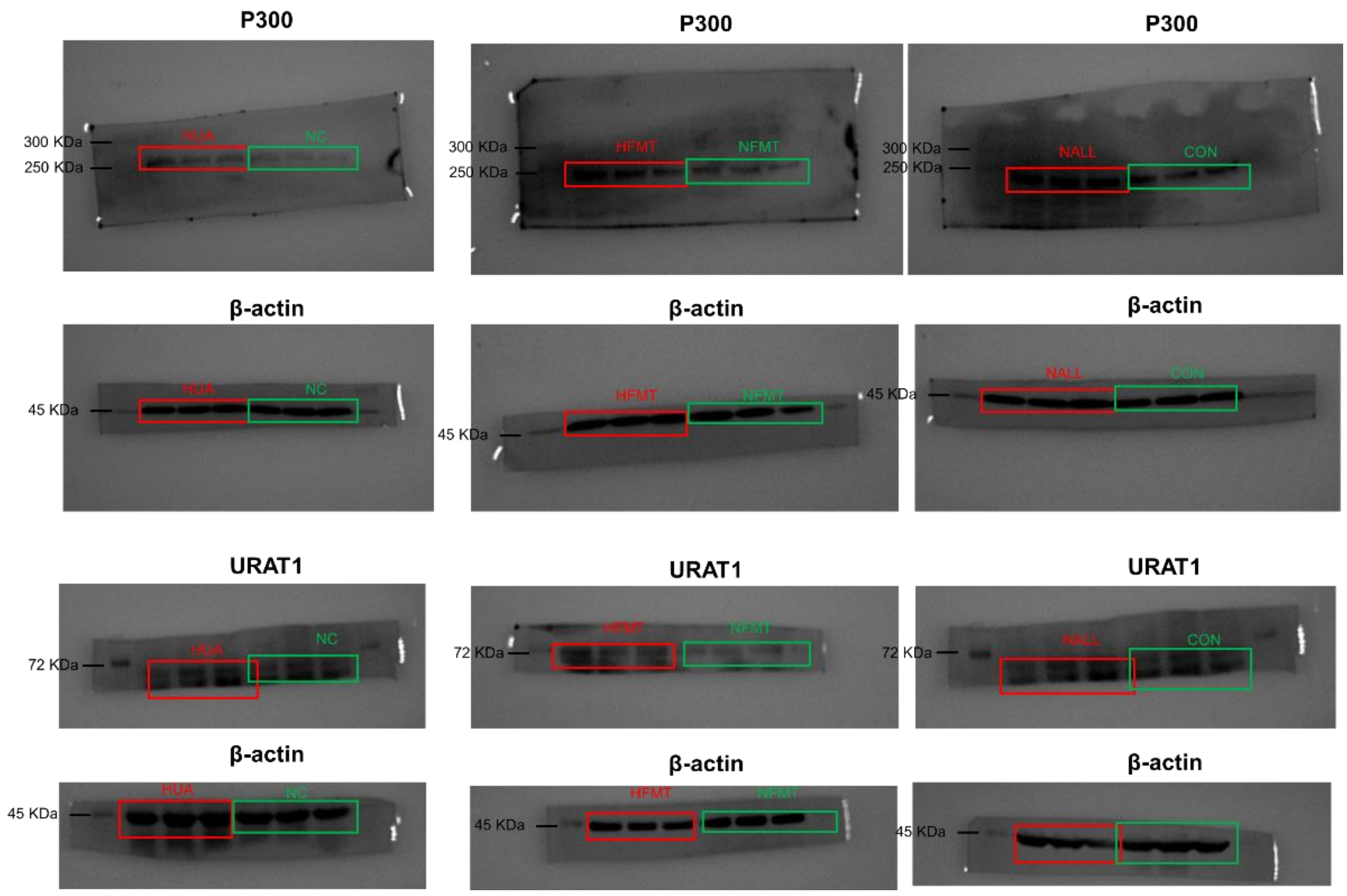

Fig. S6 Uncropped images of blots for Fig. 8A.
